# Rab27 GTPases Distribute Extracellular Nanomaps for Invasive Growth and Metastasis: Implications for Prognosis and Treatment

**DOI:** 10.3390/ijms14059883

**Published:** 2013-05-10

**Authors:** An Hendrix, Olivier De Wever

**Affiliations:** Laboratory of Experimental Cancer Research, Department of Radiation Oncology and Experimental Cancer Research, Ghent University Hospital, De Pintelaan 185, 9000 Ghent, Belgium; E-Mail: Olivier.dewever@ugent.be

**Keywords:** Rab GTPase, exosome, cancer, extracellular vesicle

## Abstract

The Rab27 family of small GTPases regulates exocytosis of distinct vesicle types including multivesicular endosomes, which results in the release of exosomes. Exosomes are nanometer-sized membrane vesicles that enclose soluble factors such as proteins and nucleic acids within a lipid bilayer and can travel toward distant tissues to influence multiple aspects of cell behavior. In our view that tumors are endocrine organs producing exosomes, Rab27 GTPases and their effector proteins are critical determinants for invasive growth and metastasis. Rab27 proteins and their effectors may serve as prognostic biomarkers or as targets for patient-tailored therapy.

## 1. Introduction

Tumors are ecosystems characterized by an intense communication between cancer cells and stromal cells [1]. These heterotypic interactions are a prerequisite for invasive growth and the final stage of multi-step tumor progression—metastasis [2]. Secretory products from both cancer cells and stromal cells are part of an endocrine signaling network that initiates the formation of a metastatic niche, a landing dock for future metastatic colonization [3]. Subpopulations of cancer stem cells educate target organs to resemble the primary tumor environment and recruit precursor cells from reservoir sites such as bone marrow [4]. Desmoplasia consisting of carcinoma-associated fibroblasts and extracellular matrix deposition is a morphological evidence of stroma participation in the primary tumor and metastatic niches [5].

Communication in the tumor ecosystem involves direct cell-cell interactions, but also the release of single molecules (growth factors, cytokines, chemokines, proteases, matrix molecules) and membrane vesicles that enclose soluble factors within a lipid bilayer [6]. An example of single molecule communication is the recruitment of mesenchymal stem cells from the bone marrow reservoir to the primary tumor and the release of neuregulin-1 to support cancer cell fitness [7]. One type of extracellular membrane vesicles that receive increased attention nowadays are exosomes, *i.e.*, nanomaps or small membrane vesicles which contain numerous proteins, lipids and even nucleic acids, and can travel to distant tissues to influence multiple aspects of cell behavior, including metastatic niche preparation [8]. Rab GTPases recruit specific effector proteins and control intracellular vesicle transport, including plasma membrane delivery and fusion of a number of different secretory vesicle types [9]. The Rab27 family of small GTPases is one important regulator of exosome release [10] and is critically involved in breast cancer progression [11–13]. Experimental data from our group and others are reviewed with a focus on the secretory Rab27 family of small GTPases and their implications in cancer progression.

## 2. Rab27 GTPases Guide Vesicle Exocytosis

Exocytosis involves vesicle transport through the cytoskeleton, the tethering and docking of vesicles at exocytic active plasma membrane sites, followed by membrane fusion between the vesicle and the plasma membrane. This process results in the secretion of vesicle contents into the extracellular environment. One group of proteins central in defining these exocytic events is the family of small Rab27 GTPases consisting of two members, Rab27A and Rab27B [14]. Posttranslational prenylation of carboxy-terminal cysteine residues allows these Rabs to reversibly localize to vesicle membranes. The switch between GTP- and GDP-bound forms (promoted by specific guanine nucleotide exchange factors and GTPase-activating proteins, respectively) is crucial for the function of small GTPases [9]. In their GTP-bound form, Rab27 proteins bind effector proteins that act during vesicle formation, movement, tethering, and fusion, with each pathway having its own unique set of effectors [15]. Eleven Rab27-specific effectors have been identified during vesicle movement [16]. The spatiotemporal recruitment of these effectors is the principal way by which Rab27 GTPases control the efficiency and the specificity of exocytosis of different vesicle types in a cell type-specific manner [15]. Rab27 GTPases are widely expressed, whereas the expression of their effectors is suggested to be much more restricted, opening a potential window of opportunity for selective targeting [17,18].

**Figure 1:**
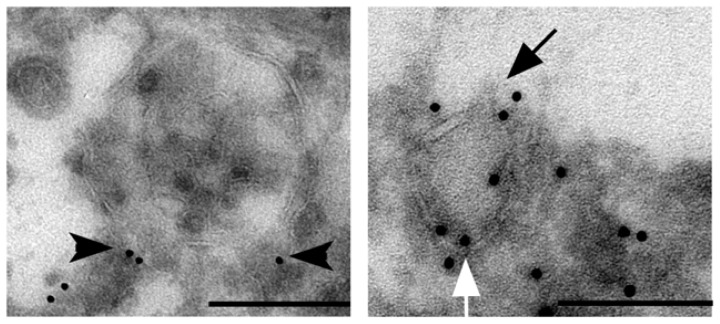
Immuno-electron microscopy of MCF-7 breast cancer cells expressing the fusion protein GFP-Rab27B. Electron micrographs of ultrathin cryosections of MCF-7 GFP-Rab27B cells grown on plastic substrate. Rab27B vesicles were immunogold-labeled with anti-GFP antibodies (10 nm gold). **Left panel**: multivesicular endosome (arrowhead: Rab27B localization on the membrane surface of a multivesicular endosome; scale bar: 500 nm). **Right panel**: secretory granule-like vesicle (white arrow: Rab27B localization on the membrane surface of a secretory vesicle; black arrow: point of exocytosis in the extracellular environment; scale bar: 100 nm).

Rab27 family members bind to the surface of distinct vesicle types including lysosome-related organelles such as melanosomes in melanocytes and lytic granules in cytotoxic T lymphocytes, secretory granules in mast cells and zymogen granules in pancreatic cells [8]. Recently, Rab27 GTPases have been identified on the cytoplasmic side of the lipid bilayer of multivesicular endosomes (MVEs) in HeLa cells [10]. MVEs are complex intracellular organelles that are formed by the invagination of the limiting membrane of an endosomal vesicle such that many small intra-endosomal vesicles are formed. Recruitment to the cell periphery and fusion of these MVEs with the plasma membrane results in the release of the internal vesicles, termed exosomes [8]. In HeLa cells, spontaneous secretion of exosomes is strongly decreased when expression of Rab27 GTPases is reduced by short hairpin RNA targeting. In addition, mouse dendritic cells deficient in both Rab27A and Rab27B secrete half the number of exosomes compared to their wild type counterparts [10]. Inhibitory RNA targeting of Rab27 GTPases in human MDA MB-231 and mouse 4T1 breast cancer cells resulted in decreased exosome numbers in culture media [13,19]. This indicates that Rab27 GTPases are general regulators of MVE transport to the plasma membrane. Vesicle localization studies in HeLa cells using confocal microscopy demonstrated that Rab27B mediates the transfer of MVEs from microtubules to the actin-rich cortex and their retention at the cell periphery, whereas Rab27A is required for the docking to the plasma membrane [10]. Rab27A depletion decreases exosome release as well as the secretion of exosome-independent proteins [13]. Immuno-electron microscopy of MCF-7 breast cancer cells demonstrates that Rab27B is localized on the membrane surface of MVE, but also smaller secretory granule-like structures (Figure 1), which suggests that in cancer cells Rab27 small GTPases are not restricted to the regulation of MVE secretion, but also other secretory vesicle types.

## 3. Rab27 GTPases Drive Invasive Growth and Metastasis

Rab27 GTPase-mediated vesicle exocytosis is an attractive upstream regulator candidate of invasive growth and metastasis, because vesicles can contain entire sets of pro-invasive factors, including proteins, mRNAs and miRNAs [20]. Fusion of these vesicles with the plasma membrane results in the release of single molecules and/or exosomes with the ability to modulate and determine tumor cell behavior in the local tumor ecosystem and at distant sites. A role of exosomes in distant cell-to-cell communication was revealed by delivery of exosome-packaged biological active mRNA and miRNA from a donor cell to a recipient cell, influencing the latter’s RNA expression, proteome and functions [21].

Work from our group demonstrated that Rab27B promotes invasive growth and metastasis of estrogen receptor (ER)-positive breast cancer cells [11]. Breast cancer cells in which Rab27B was overexpressed formed cellular extensions and a spread morphology and had a significant increased ability to invade Matrigel and native type I collagen substrates. In addition, Rab27B enhanced proliferation under limiting serum concentrations. Rab27B stimulated metabolic reprogramming from oxidative phosphorylation towards aerobic glycolysis in ER-positive breast cancer cells which was accompanied by acidification of the tumor environment [22]. In orthotopic xenograft models Rab27B promoted invasive tumor growth as evidenced by increased tumor volume and weight, and massive infiltration of the breast cancer cells into the abdominal skeletal muscles. Anchorage-independent metastatic cancer cells were present both as single cells or aggregates in the peritoneal cavity [6]. Mass spectrometric identification of proteins residing in intracellular Rab27B vesicles revealed different exosome markers including the tetraspanins CD9, CD63 and CD81, and the heat shock proteins HSP70 and HSP90α (Table 1) [11].

Rab27B overexpression in breast cancer cells resulted in a four-fold increase in the release of HSP90α. HSP90α is a molecular chaperone with intracellular and extracellular (amongst which exosomal and non-exosomal associated) functions. Extracellular HSP90α can be present as a soluble protein or exosome-surface bound protein. The chaperone can be secreted by non-conventional exocytosis which involves a *C*-terminal EEVD motif that interacts with proteins containing tetratricopeptide repeat domains and phosphorylation of residue Thr-90 [23]. Flow cytometry analysis located HSP90α at the membrane surface of exosomes [24]. Also, acidic extracellular pH has been identified as a stimulus of vesicle rupture resulting in the subsequent release of the content [25]. This could further contribute to the presence of free HSP90α in the extracellular environment. One reported client protein of extracellular HSP90α is MMP-2, a matrix metalloprotease that requires chaperoning for its activity [26]. A complex of co-chaperones (HSP70, Hop, HSP40, and p23) is present outside of breast cancer cells and co-immunoprecipitates with HSP90α *in vitro* and in breast cancer conditioned media [27]. These co-chaperones also increase the association of HSP90α and MMP-2 and enhance HSP90α-mediated activation of MMP-2. These findings support a model in which MMP-2 activation by an extracellular co-chaperone complex mediated by HSP90α increases breast cancer cell migration and invasion. Probably, the synergistic effect of this extracellular chaperone in the primary tumor and exosomes in the metastatic sites is fundamental for the observed functional effects, *i.e*., invasive growth and hemorrhagic ascites, in our xenograft models. Future research aims to unravel the substantial contribution of different Rab27B-steered secretory pathways to the development of an extracellular signaling network for cancer progression. One approach involves the complete determination of exosome-enriched fractions and exosome-depleted conditioned media of control *versus* Rab27B overexpressing breast cancer cells. Furthermore, in three different ER-positive breast cancer models we demonstrated that Rab27A was not able to induce proliferation and invasion in collagen type I matrices. Since Rab27A and Rab27B share 70% homology and have the potential to interact with a same set of effector proteins this raises an important cell biological question for further investigation.

Rab27A was identified as a driver gene that provides growth advantage during melanoma progression [28]. This study made use of a computational framework that integrates chromosomal copy number and gene expression to detect driver genes located in regions that are amplified or deleted in tumors. Also, Rab27A inhibition in melanoma cell lines reduced primary tumor growth and development of lung metastasis in xenograft mouse models [29]. This effect was due to a combination of impaired secretion of pro-angiogenic factors and decreased secretion of exosomes that educate and recruit bone marrow derived cells to establish a pre-metastatic niche. Combined Rab27A dependent-release of MMP-9 and exosomes further contributes to the mobilization of a pro-tumoral neutrophil population and supports growth of a mouse mammary tumor and its metastasis in lung [13].

In bladder cancer aberrant expression of not only several Rabs, but also Rab effectors, GAPs en GEFs was illustrated [30]. Indeed, we recently identified a role for vacuolar H^+^ (V)-ATPase proton pump in Rab27B-dependent exocytosis and demonstrated that Rab27B expression stimulated V-ATPase expression suggesting that increased secretory GTPase activity induces expression of components necessary to support and steer GTPase regulated vesicle transport [31]. Pharmacological targeting of V-ATPase activity by nanomolar concentrations of the macrolide antibiotic bafilomycin A1 resulted in a four-fold decreased release of HSP90α in the conditioned medium by breast cancer cells. In agreement, by using two different genetic approaches, the membrane-bound V_0_ sector of V-ATPase has been proposed to regulate MVE secretion at the apical plasma membrane in a *Caenorhabditis elegans* model [32]. Our results point to a similar regulatory mechanism in mammalian cells.

## 4. Clinical Assessment of Experimentally Validated Pro-Invasive Rab27 GTPases

Breast cancer heterogeneity is classified into molecular and histopathologic subtypes based predominantly on the expression of hormone and growth factor receptors—namely ER, progesterone receptor (PR), and HER2. Patients with a triple-negative breast tumor (*i.e*., ER^−^/PR^−^/HER2^−^) are at the greatest risk of early recurrence. ER-positive breast tumors are the most prevalent. These tumors are often differentiated and associated with good prognosis, yet a significant number of patients experience disease relapse even 10 to 15 years after their initial diagnosis and cancer management [33]. Today’s tumor classification has some correlation with patient outcome, however it is difficult to accurately predict which patients will relapse. Therefore, we need to find more accurate, sensitive and easy accessible predictors of metastatic disease [12].

The observation that Rab27B is a key factor for increased invasiveness, tumor size, and metastasis of various ER-positive breast cancer cell lines *in vitro* and *in vivo* suggests that this small Rab GTPase provides an important marker in the signature of ER-positive breast cancers with poor prognosis [12]. Fluorescence in situ hybridization on primary human breast cancer samples detected no amplification of the *Rab27B* gene [11]. But quantitative real-time PCR revealed a 10-fold increase in Rab27B mRNA expression in breast tumor tissue (*n* = 20) compared with normal tissue (*n* = 5) suggesting a higher Rab27B promoter activity or higher posttranscriptional stabilization of Rab27B mRNA. In addition, Rab27B mRNA levels were significantly higher in ER-positive primary breast cancer with positive nodal status. Rab27A mRNA levels did not significantly differ. Primary breast cancer specimens (*n* = 59) were analyzed by immunohistochemistry using an anti-Rab27B specific polyclonal antibody. In ER-positive breast cancer a significant correlation was found among high Rab27B expression, nodal status and tumor grade [11]. Rab27B is a well-defined biological marker with the high potential to improve ER-positive breast cancer subclassification. Independently, Rab27B expression analysis on a cohort of 221 primary breast cancer samples confirmed increased Rab27B expression in poor-prognosis breast cancer and identified Rab27B as an independent risk factor for survival [34]. Immunohistochemical assessment of Rab27 GTPases in 148 primary hepatocellular carcinoma samples demonstrated that presence of Rab27A or Rab27B significantly associated with reduced overall survival [35]. The current correlations between Rab27 GTPases and cancer involve the upregulation of mRNA and/or protein. Although loss-of-function mutations in the Rab27A gene are well-described and known to cause Griscelli type 2 syndrome, it is unclear whether mutations in Rab27 genes exist and play a role in cancer biology. Regulation of Rab expression by miRNAs has been reported and miRNA124a has been identified as a negative regulator of Rab27A expression [36].

In future long-term vision, a prognosis marker may serve as a therapeutic target; widely used examples are anti-hormone therapy based on ER positivity and Trastuzumab treatment based on HER2 positivity. A similar approach is feasible for Rab27 GTPases using effector specific antagonists to block exosome release with the aim to control metastasis. Two potential candidates are the Rab27-specific effector proteins slp4 and slac2b that are involved in the regulation of MVE transport [10]. V-ATPase expression and activity controls Rab27B-induced collagen type I invasion, cell cycle progression and invasive growth [31]. Poor prognosis ER-positive primary breast tumors expressing high levels of Rab27B also expressed multiple V-ATPase subunits and showed a strong cytoplasmic and peripheral V-ATPase expression. Clinically, proton pump inhibitors (PPIs), such as esomeprazole, have been largely and successfully used for the treatment of peptic ulcer diseases. PPIs target (H1/K1)-ATPases localized within the intracellular secretory lumen of gastric parietal cells. Most importantly, PPIs also inhibit the activity of V-ATPases, and thus are potential candidates to block Rab27B-mediated exosome release.

Exosomes are released into the interstitial fluid and may accumulate into biofluids like blood, and urine, and are currently emerging as potential non-invasive biomarkers for cancer staging. Multicenter studies for biomarker validation would require standardized exosome preparations from these biofluids to minimize variability generated by sample collections and isolation techniques. Pre-analytical steps such as agitation during transportation, time delay to the first centrifugation step and the centrifugation protocol are potential sources of variability [37]. Thus, identification and quantitative characterization of nanometer-sized exosomes remains challenging. Direct measurements in the biofluid would be of benefit to avoid manipulation artefacts. Furthermore, to prevent sample contamination and enable high throughput analysis, these measurements are preferentially performed in inexpensive disposable sample holders. One promising approach combines on-chip microfiltration and miniaturized nuclear magnetic resonance to enable quantitative detection of exosomes labeled with target-specific magnetic nanoparticles [38]. We developed a low-cost disposable microfluidic chip with integrated light sheet illumination for high-throughput exosome staging in cancer patients [39]. Light sheet illumination enables on-chip detection of exosomes that are fluorescently labeled directly in biofluids without pre-processing steps.

## 5. Conclusions

Despite many advances, cure rates for advanced cancer remain low. There is an imperative need to identify new targets to control metastatic cancer. We consider cancer as an endocrine organ that releases exosomes affecting the host at a systemic level and particularly at the preparation of metastatic niches. The traits that a cancer acquires to successfully grow and metastasize to distant sites are at least partly regulated by aberrant vesicular transport and its effectors are major contributors to this regulatory process. Future experiments are expected to define selective and specific functions for Rab27 effectors in exosome release, as well as establish potential opportunities for immunohistochemical and blood-based prognostic biomarkers and therapeutic intervention for the treatment of cancer.

## Figures and Tables

**Table 1:** Mass spectrometric identification of exosome markers in purified intracellular Rab27B vesicle fractions.

Protein identity	Matched peptides (#)	Sequence coverage (%)
Tetraspanins		
CD9	3	29.6
CD63	1	14.2
CD81	3	21.5

Heat shock proteins		
HSP70	21	44.3
HSP90 alpha	13	32.9
